# Comparison of gut microbiota between immigrant and native populations of the Silver-eared Mesia (*Leiothrix argentauris*) living in mining area

**DOI:** 10.3389/fmicb.2023.1076523

**Published:** 2023-01-24

**Authors:** Tianlong Zhou, Shilong Liu, Aiwu Jiang

**Affiliations:** Guangxi Key Laboratory of Forest Ecology and Conservation, College of Forestry, Guangxi University, Nanning, China

**Keywords:** gut microbiota, *Leiothrix argentauris*, trace element, immigrant subspecies, native subspecies

## Abstract

The complex gut bacterial communities have a major impact on organismal health. However, knowledge of the effects of habitat change on the gut microbiota of wild birds is limited. In this study, we characterized the gut microbiota of two different subspecies of the Silver-eared Mesia (*Leiothrix argentauris*), the native subspecies (*L. a. rubrogularis*) and immigrant subspecies (*L. a. vernayi*), using 16S rRNA gene high-throughput sequencing. These two subspecies live in a trace metal-contaminated area, and *L. a. vernayi* was trafficked. They are an excellent system for studying how the gut microbiome of wild animal changes when they move to new habitats. We hypothesized that the immigrant subspecies would develop the same adaptations as the native subspecies in response to habitat changes. The results showed that there were no significant differences in the composition, diversity, or functional metabolism of gut microbiota between native and immigrant subspecies under the combined action of similar influencing factors (the *p* values of all analyses of variance >0.05). In addition, the composition and functional metabolism of gut microbiota in two subspecies showed adaptation against trace metal damage. Linear discriminant analysis effect size (LEfSe) analysis revealed that *Massilia* in the intestinal microbiota of immigrant subspecies was significantly higher than that of native subspecies, suggesting that immigrant subspecies suffered habitat change. Finally, we found that these two subspecies living in the mining area had an extremely high proportion of pathogenic bacteria in their gut microbiota (about 90%), much higher than in other species (about 50%) living in wild environment. Our results revealed the adaptation of intestinal microbiota of immigrant Silver-eared Mesias under heavy metals stress, which would provide guidance for biodiversity conservation and pollution management in mining area.

## Introduction

Vertebrates exhibit a highly complex symbiotic relationship with their gut microbiota (McFall-Ngai et al., 2013). The gut microbiota is conducive to metabolism, immunity, behavior, and development of the organisms, with positive implications on host health (McFall-Ngai et al., 2013; Youngblut et al., 2019). For instance, the gut microbiome can regulate nutrient absorption efficiency, influence external temperature, and control metabolic rate (Jumpertz et al., 2011; Chevalier et al., 2015). Besides, some specific microorganisms can enhance the resistance of the host to toxicity (Kohl et al., 2014). In turn, the hosts provide a nutrient-rich, stable habitat for the gut microbiome (Bodawatta et al., 2022a). Hosts and their gut microbiota have shaped a complex symbiotic relationship.

The animal’s gut microbiome is derived primarily from the environment (Candela et al., 2012). As a result, the gut microbiome is an unstable element, constantly varying in response to external environment changes (Candela et al., 2012). For example, animals can acquire new gut microbes from the environment (Hehemann et al., 2010). The relative abundance of gut microbes can be reconfigured according to various environmental factors (Candela et al., 2012). The hosts’ metabolism (Fan and Pedersen, 2021), nutrition (Moszak et al., 2020), immunology (Grond et al., 2018), behavior (Morais et al., 2021), morphology (Broderick et al., 2014) and development (Gilbert et al., 2015) would be affected by gut microbiome changes. Understanding how environmental change dictates the microbiota in the intestine will facilitate the conservation and management of wildlife (Alberdi et al., 2016; Rosenberg and Zilber-Rosenberg, 2018).

Many factors cause animals to alter their habitat. These include natural migrations, such as the migration of birds and fish, as well as forced migrations, such as captivity, global warming, and animal trade. Generally, intestinal microbiota changes caused by natural migration are usually weak - most bacterial taxa are significantly unaffected (Risely et al., 2017, 2018). Conversely, wildlife will lose their native microbiome and reduce the alpha diversity of gut microbiome when moving to captivity (Alberdi et al., 2021; Dallas and Warne, 2022). Forced migrations caused by climate warming often result in gut microbiota variations due to diet changes (Chen et al., 2022a,b). The illegal trade of animals usually ends in captivities or the release of wildlife to new habitats (Ni et al., 2020). However, previous research always focused on the gut microbiota of animals in captivities (Alberdi et al., 2021; Dallas and Warne, 2022). The gut microbiome of illegally traded wildlife released into new natural habitats is poorly known.

The Silver-eared Mesia (*Leiothrix argentauris*), of least concern threat-status (IUCN), is a resident bird and distributed mainly in forests around China and India, as well as in Sumatra^1^. This bird does not show distinct sexual dimorphism. Its feathers are brightly colored, varying in different subspecies. Silver-eared Mesia is ubiquitous in international live-bird trade because of its unique characteristics (Li and Jiang, 2014; Eaton et al., 2015). This eventually leads to the traded Silver-eared Mesias being raised in captivity or introduced into natural habitats where other native populations live. In addition, the immigrant and native population of Silver-eared Mesia may live in the same flock because of their gregarious habits. Hence, Silver-eared Mesia is an excellent model to study how the gut microbiota of wildlife would change after moving to a new habitat by international trade.

In this study, we reported the composition of gut microbiota in the two subspecies of Silver-eared Mesia, including native (*L. a. rubrogularis*) and immigrant subspecies (*L. a. vernayi*), at a mine area in Southern China. The overall goal was to compare the composition, diversity, and functional characteristics of the gut microbiota of both native (*L. a. rubrogularis*) and immigrant (*L. a. vernayi*) populations. In addition, we also analyzed the intestinal microbiome under trace element stress to explore whether the immigrant population enhanced adaptability to the new environment for the host.

## Materials and methods

### Ethical guidelines

All samples of feathers and cloacal swabs of Silver-eared Mesias were obtained under the permission of Chongzuo Forestry Department (2018. 1), and Animal Ethics Committee, Guangxi University (GXU2018-039), China, and procedures followed the laws of the People’s Republic of China.

### Study area overview

We conducted this study in a mine tailing (22°58′15″N, 107°17′28″E) in the Daxin County of Chongzuo Prefecture in Guangxi, Zhuang Autonomous Region, Southern China. The French began mining here more than 100 years ago. However, due to environmental pollution and the depletion of mineral resources, the mine was closed in 2001 (He et al., 2020). The region has a subtropical Marine monsoon climate, with an average annual precipitation of 1348.8 mm. Due to water leaching and surface runoff, the downstream rivers and soil are seriously polluted by trace elements. Cadmium levels in the soil were 11.3 times higher than the recommended limit set in 2000 (Pan et al., 2020).

### Study species

There are two subspecies of the Silver-eared Mesia in this area. One is a native subspecies, *L. a. rubrogularis*, which is naturally distributed in the studied area (Jiang A. et al., 2021). Another subspecies, *L. a. vernayi*, which is distributed initially mainly in Myanmar and Southwestern China, has recently been observed in field monitoring. The plumage characteristics of the two subspecies are similar. The only apparent difference is that *L. a. rubrogularis* has a red collar and base coverings of upper tail, while *L. a. vernayi* is orange. The diet of Silver-eared Mesia is uniformly omnivorous, feeding on insects and their larvae, fruits, and seeds.

### Sample collection

We captured the two subspecies living in the forests around the tailing pond using mist nets in January 2022. We used passive methods (no birdsong playback), placing nets from dawn to dusk and patrolling them at least once per hour. We eventually captured 19 Silver-eared Mesias (5 *L. a. rubrogularis* and 14 *L. a. vernayi*). Once birds are captured, we cleaned the outside of the cloaca with an alcohol pad, inserted a sterile flocking swab (Puritan 25-3,316-U Ultra Flocked Swab, United States) fully into the cloaca, turned for 3–5 s, and preserved the swab in RNAlater (Qiagen, Hilden, Germany). Then, samples were immediately placed in sterile vials, kept in a cool box in the field, and later stored at −20°C. Upon return to the laboratory, all samples were stored at −80°C until processed. Before color-mapping and release, we measured the body index of each bird (including body weight, body length, length of wing and length of tarsometatarsus). Besides, we collected each bird’s primary, secondary, chest and tail feathers to measure trace element levels. The measurement method was described in detail in the Supplementary materal.

### DNA extraction, PCR amplification, and amplicon sequencing

Total genome DNA from samples was extracted using cetyltrimethylammonium bromide (CTAB) method. DNA concentration and purity were monitored on 1% agarose gel. According to the concentration, DNA was diluted to 1 ng/μL using sterile water.

PCR amplification of the V3–V4 hypervariable region of bacterial 16S rRNA genes was performed using the bacterial-specific forward primer 341F (5′-CCTAYGGGRBGCASCAG-3′) and reverse primer 805R (5′-GGACTACNNGGGTATCTAAT-3′) with the barcode. All PCR reactions were carried out with 15 μl of Phusion® High-Fidelity PCR Master Mix (New England Biolabs); 2 μM of forward and reverse primers, and about 10 ng template DNA. Thermal cycling consisted of initial denaturation at 98°C for 1 min, followed by 30 cycles of denaturation at 98°C for 10 s, annealing at 50°C for 30 s, and elongation at 72°C for 30 s, followed by a final extension of 5 min at 72°C. Mix same volume of 1X TAE buffer with PCR products and operate electrophoresis on 2% agarose gel for detection. PCR products was mixed in equidensity ratios. Then, mixture PCR products was purified with Qiagen Gel Extraction Kit (Qiagen, Germany).

Sequencing libraries were generated using TruSeq® DNA PCR-Free Sample Preparation Kit (Illumina, United States) following manufacturer’s recommendations and index codes were added. The library quality was assessed on the Qubit@ 2.0 Fluorometer (Thermo Scientific). At last, the library was sequenced on an Illumina NovaSeq platform and 250 bp paired-end reads were generated (a total of 19 samples, 5 *L. a. rubrogularis*, and 14 *L. a. vernayi*). Raw sequences obtained in this study are available through the National Center for Biotechnology Information (NCBI) database (accession number PRJNA853520).

### 16S rRNA gene amplicon sequencing analysis and processing

Microbiome bioinformatics analysis were performed with the QIIME 2 (2022.2) process (Bolyen et al., 2019). With reference to the review by Knight et al. (2018), we made slightly modified based on the official tutorial^2^. In short, paired-end reads were merged using ‘vsearch join-pairs’ and quality filtered using ‘quality-filter q-score-joined’ (Bokulich et al., 2013) within QIIME2 after trimming the barcode and primer sequences from the reads. Next, sequences were quality filtered and denoised using the Deblur workflow (Amir et al., 2017). All amplicon sequence variants (ASVs) were aligned with mafft (Katoh et al., 2002). Taxonomy was assigned to ASVs using ‘feature-classifier classify-sklearn’ plugin against the pre-trained Naive Bayes classifier (Bokulich et al., 2018) [based on SILVA v138 database (Quast et al., 2012)]. Then, the ASVs containing less than 0.1% of total sequences across all samples, any contaminating mitochondrial and chloroplast sequences and sequences not assigned to phylum were filtered out from the denoising results. Next, ‘fragment-insertion sepp’ (Mirarab et al., 2012) is used to generate our phylogenetic tree by inserting short molecular sequences into an existing phylogenetic tree and aligning each short molecular sequence to the aligment of the full-length sequences, then that alignment is used to find the optimal location in the phylogenetic tree for querying sequences. Finally, to minimize the difference of sequencing depth across samples, data was rarefied to a sampling depth of 17,400 reads per sample for the downstream diversity analysis.

### Metagenomic pathway prediction by PICRUSt2

The 16S rRNA gene sequence of the sample was mapped to KEGG^3^ database for annotation, and the abundance of metabolic pathways was predicted based on Phylogenetic Investigation of Communities by Reconstruction of Unobserved States (PICRUSt2) (Douglas et al., 2020). R software (v4.1.2) was utilized for statistical analyses and visualization of the identified pathways. Correlations between samples were visualized using the pheatmap package. Linear discriminant analysis effect size (LEfSe) analysis was used to detect the differentially abundant metabolic pathways between *L. a. rubrogularis* and *L. a. vernayi*.

### Statistical analysis

Statistical analysis was completed in R v4.1.2 (R Core Team, 2021). Alpha diversity analysis was carried out using the “picante” package (v1.8.2) (Kembel et al., 2010). Differences in Alpha diversity between *L. a. rubrogularis* and *L. a. vernayi* were evaluated by one-way ANOVA (Jobson, 1991), and all *p* values were corrected *via* the false discovery rate (FDR) correction. Rarefaction curves were generated by “ggplot2” package (v3.3.5) (Wickham, 2016). The ASV-level ranked abundance curves were generated to compare the richness and evenness of ASVs among samples. Venn diagram was generated to visualize the shared and unique ASVs among groups using the R package “VennDiagram” (v1.7.3) (Hanbo, 2022), based on the occurrence of ASVs across samples/groups regardless of their relative abundance. Beta diversity analysis was performed to investigate the structural variation in microbial communities across samples using weighted and unweighted UniFrac distance metrics (Lozupone et al., 2011) and visualized *via* principal coordinate analysis (PCoA) (Ramette, 2007). Differences in the UniFrac distances for pairwise comparisons among groups were determined by Anosim test with 999 permutations using “vegan” package (v2.5–7) (Jari et al., 2020). The taxonomic composition of each sample was visualized as a stacked bar plot at the phylum, family and genus level with the “ggplot2” package (v3.3.5) (Wickham, 2016). The circles plot of *L. a. rubrogularis* and *L. a. vernayi* were generated by Circos^4^ (Krzywinski et al., 2009). Correlations between samples were visualized using the pheatmap package (v1.0.12) (Raivo, 2019). Linear discriminant analysis effect size (LEfSe) was performed to detect differentially abundant taxa across groups using the default parameters (Segata et al., 2011).

## Results

### Sequencing quality and ASVs distribution

**Figure 1:**
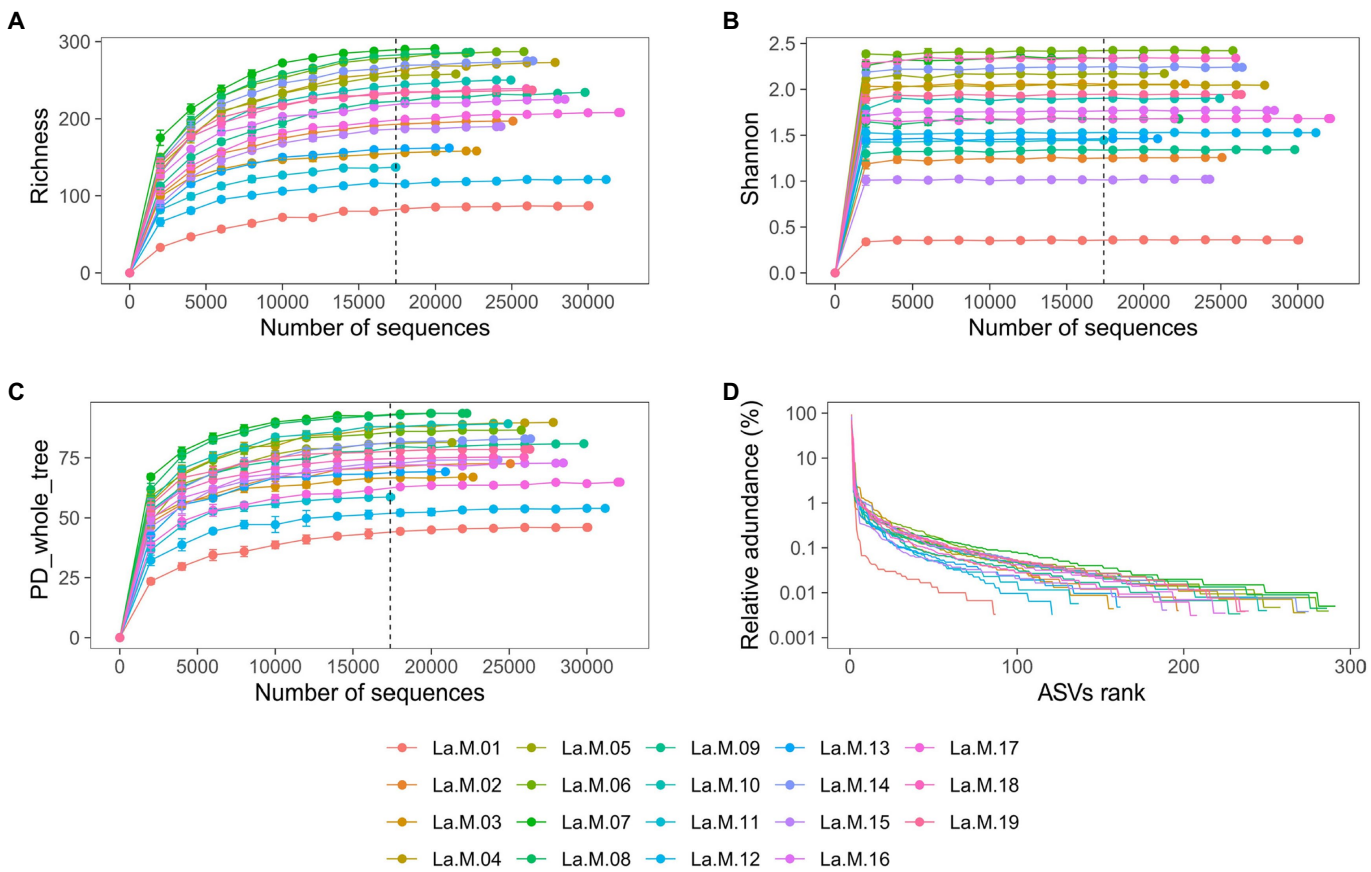
Sequencing depth analysis and ASVs distribution of gut microbiota in *L. a. rubrogularis* and *L. a. vernayi*. Rarefaction curves of gut microbiota of all samples based on Illumina MiSeq sequencing. Horizontal axis: the amount of effective sequencing data; vertical axis: **(A)** the observed number of operational taxonomic units (richness index); **(B)** Shannon index; **(C)** and PD_whole_tree. **(D)** Rank abundance curve of gut microbiota of *Leiothrix argentauris*. Horizontal axis: the number of ASVs according to the abundance, from high to low. Vertical axis: the abundance of ASVs. The larger the span curve on the horizontal axis, the higher the species richness. The smoother the curve on the vertical axis, the more even the species distribution.

After strict quality filtering, a total of 482,768 16S rRNA gene reads were obtained, with an average of 25,409 ± 3,882 sequences per sample (median: 25785), from which 501 ASVs were identified to be from gut microbiotas of 19 Silver-eared Mesias (*L. a. rubrogularis* = 462, *L. a. vernayi* = 501). The rank abundance and rarefaction curves supported the adequacy of the sequencing depth, indicating that almost all the bacterial species were found in intestinal samples (Figures 1A–D). A total of 92.22% of the ASVs were shared among the two groups, with unique ASVs entirely being present in the *L. a. vernayi* (7.78%). All ASVs can be detected in *L. a. vernayi* (Supplementary Figure S1).

### Characteristics of gut microbial diversity of *L. a. rubrogularis* and *L. a. vernayi*

**Figure 2:**
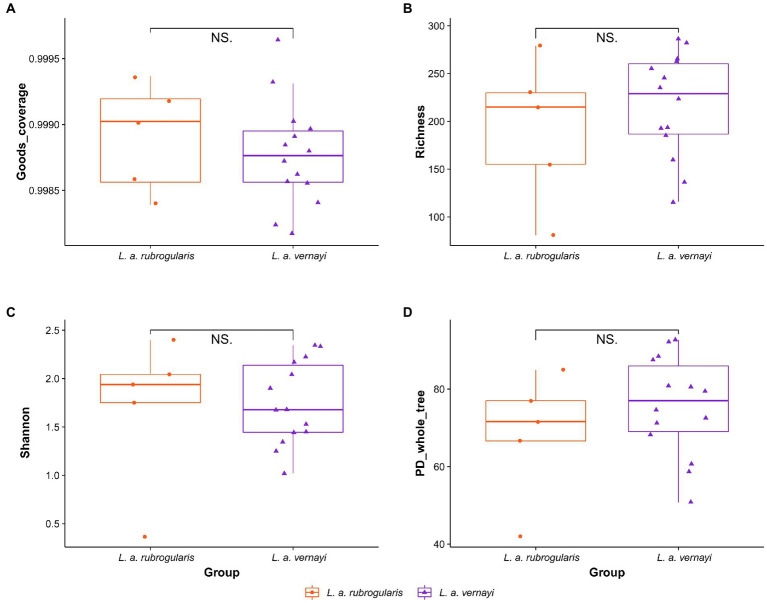
Alpha diversity of gut microbiota in the *L. a. rubrogularis* and *L. a. vernayi*. **(A)** Good’s coverage; **(B)** Richness index; **(C)** Shannon index; **(D)** PD_whole_tree. “NS.” showed no significant difference between the two groups (*p* > 0.05).

To evaluate the differences in community richness and diversity among the subspecies, the effective sequences were aligned to calculate the Good’s coverage, Richness, Shannon, and PD_whole_tree indices. Good’s coverage estimates of each sample ranged from 99.82 to 99.97%, suggesting excellent coverage (Figure 2A). Measurements of gut community alpha diversity did not vary significantly between *L. a. rubrogularis* and *L. a. vernayi* (based on one-way ANOVA, Richness index, value of *p* = 0.4350; Shannon index, value of *p* = 0.876; PD_whole_tree, value of *p* = 0.329). Thus, the diversity of intestinal microbiota between *L. a. rubrogularis* and *L. a. vernayi* was similar (Figures 2B–D).

**Figure 3:**
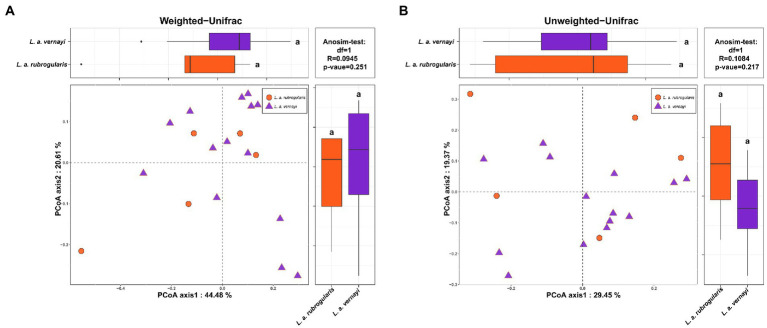
Beta diversity of gut microbiota in the *L. a. rubrogularis* and *L. a. vernayi*. **(A)** Principal coordinates analysis (PCoA) based on weighted UniFrac distance **(B)** and unweighted UniFrac distance. Wilcoxon rank-sum test of two subspecies on the first axis is shown on the upper left; Wilcoxon rank-sum test of two subspecies on the second axis is at the lower right; Anosim-test results based on bray-Curits distance between two subspecies are shown in the upper right.

Weighted and unweighted Unifrac distances are used to generate PCoA plots reflecting the gut microbial beta-diversity between individuals. No significant difference between *L. a. rubrogularis* and *L. a. vernayi* was detected no matter what weighted Unifrac distance or unweighted Unifrac distance was considered (Figures 3A,B, Anosim test: value of *p* = 0.251 and 0.217, *R* = 0.0945 and 0.1084, respectively).

### Characteristics of gut microbiota composition of *L. a. rubrogularis* and *L. a. vernayi*

**Figure 4:**
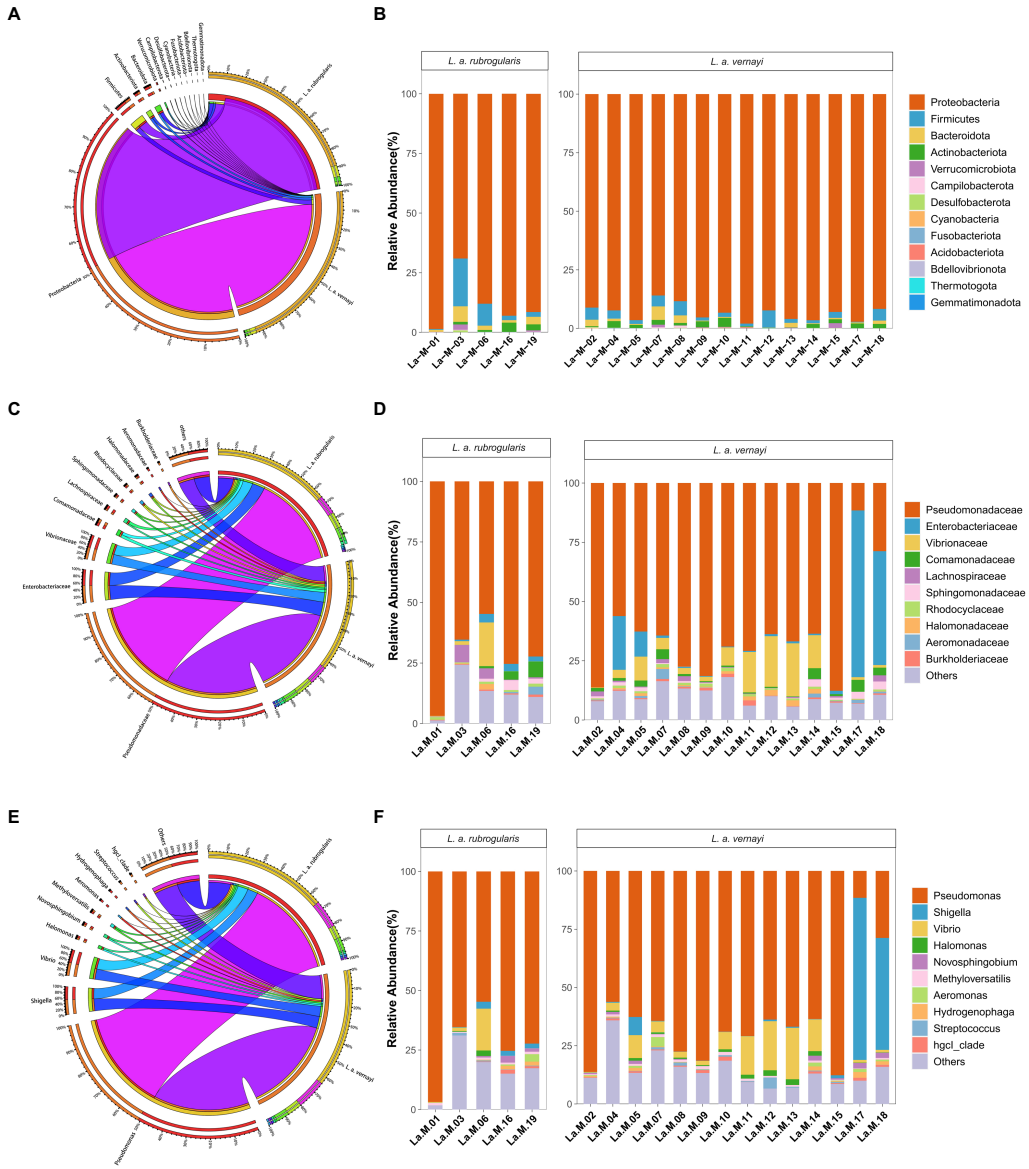
Relative abundance of the dominant taxa in the *L. a. rubrogularis* and *L. a. vernayi*. **(A)** Phylum; **(C)** family; **(E)** genus. Relative contribution of the dominant taxa in all samples. **(B)** Phylum; **(D)** family; **(F)** genus.

Nearly all reads were assignable to 13 phyla, 17 classes, 54 orders, 97 families, and 162 genera. At the phylum level, *Proteobacteria* were the most abundant bacterial phylum (88.94, 93.46%) among *L. a. rubrogularis* and *L. a. vernayi* gut communities followed by *Firmicutes* (6.05, 3.12%), *Bacteroidetes* (2.51, 1.24%), and *Actinobacteria* (1.65, 1.61%) (Figure 4A). The relative abundance of the remaining others phyla was low. All the phyla of gut microbiota in *L. a. rubrogularis* and *L. a. vernayi* were shown in Figure 4B. The relative abundance of each phylum across samples fluctuated little.

At the family level, *Pseudomonadaceae* predominated (66.04, 63.87%) among *L. a. rubrogularis* and *L. a. vernayi* gut communities followed by *Enterobacteriaceae* (8.80, 9.72%), *Vibrionaceae* (6.71, 7.48%), *Comamonadaceae* (1.96, 1.80%), *Lachnospiraceae* (1.27, 1.38%), *Sphingomonadaceae* (1.22, 1.25%), *Rhodocyclaceae* (0.87, 0.84%), *Halomonadaceae* (0.85, 0.94%), *Aeromonadaceae* (0.73, 0.63%) and *Burkholderiaceae* (0.66, 0.66%) (Figure 4C). The top 10 families of gut microbiota in *L. a. rubrogularis* and *L. a. vernayi* were shown in Figure 4D. We observed that the abundance of *Enterobacteriaceae* in two samples of *L. a. vernayi* was higher than that in other samples, and even exceeded that of *Pseudomonadaceae*.

At the genus level, *Pseudomonas* predominated (66.04, 63.87%) among *L. a. rubrogularis* and *L. a. vernayi* gut communities followed by *Shigella* (7.18, 7.90%), *Vibrio* (6.56, 7.31%), *Halomonas* (0.85, 0.94), *Novosphingobium* (0.84, 0.86), *Methyloversatilis* (0.81, 0.78%), *Aeromonas* (0.71, 0.61), *Hydrogenophaga* (0.65, 0.63%), *Streptococcus* (0.64, 0.70%), and *hgcl_clade* (0.63, 0.65%) (Figure 4E). The top 10 genera of gut microbiota in *L. a. rubrogularis* and *L. a. vernayi* were shown in Figure 4F. The abundance of *Shigella* was higher in two samples of *L. a. vernayi* than in the other samples.

### Difference analysis of the gut microflora between *L. a. rubrogularis* and *L. a. vernayi*

**Figure 5:**
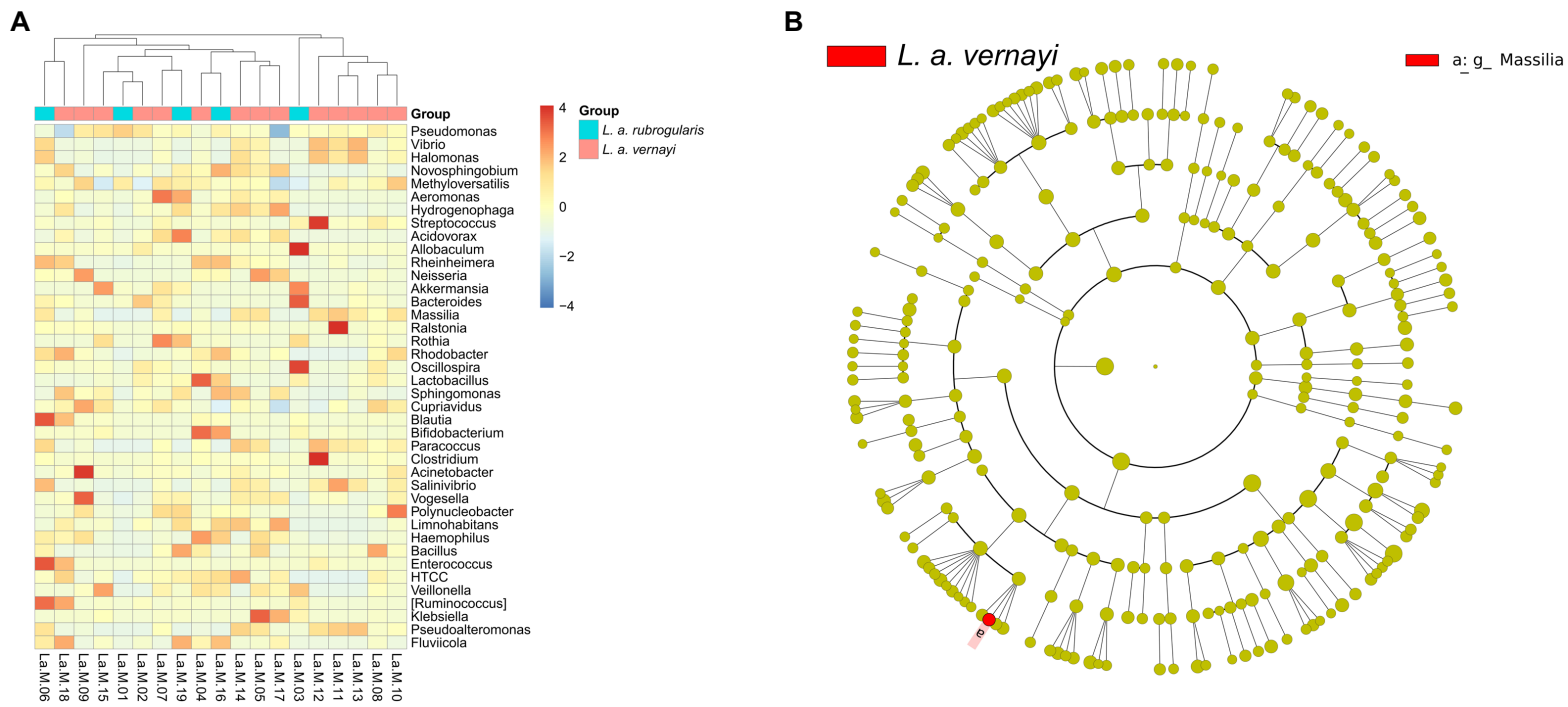
**(A)** Heatmap of the genus-level hierarchical clustering of the microbial community in *L. a. rubrogularis* and *L. a. vernayi*. **(B)** Differential microbial phylogenetic distribution was visualized by the cladogram. The taxonomic branch diagram shows the taxonomic hierarchy of the main taxa from phylum to genus (from inner ring to outer ring) in the sample community.

To further investigate whether there were differences in intestinal microbial community composition between *L. a. rubrogularis* and *L. a. vernayi*. The top 40 genera with average abundance were used to construct the heatmap of the genus-level hierarchical clustering. Although the clustering was divided into two categories, *L. a. rubrogularis* and *L. a. vernayi* were not, respectively, assigned to the two categories, and the distribution of the two groups was very dispersed (Figure 5A). LEfSe analysis was used to determine the microbial communities with significant differences in abundance between the two groups across the phylum, class, order, family, and genus levels. Interestingly, we identified only one genus taxon, *Massilia*, as having a significantly higher abundance in *L. a. vernayi* than in *L. a. rubrogularis* (LDA > 2, value of *p* < 0.05) (Figure 5B). Lefse analysis confirmed the results of genus clustering and showed that the composition of intestinal flora in *L. a. rubrogularis* and *L. a. vernayi* was basically consistent.

### Prediction and comparisons in the gut microbial metabolism pathways

**Figure 6:**
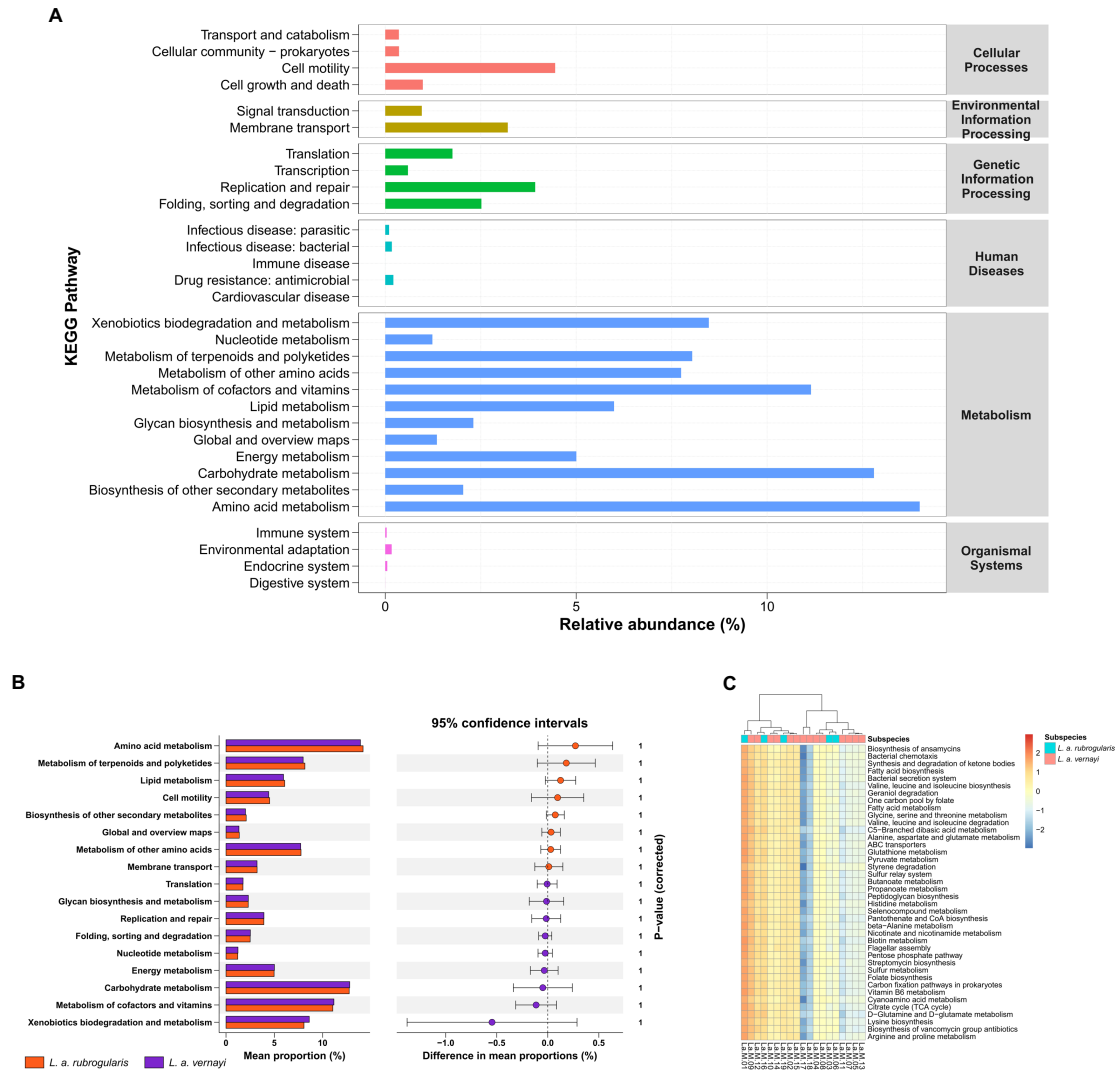
The prediction of metabolic function within the microbiome in *L. a. rubrogularis* and *L. a. vernayi* (*n* = 19). **(A)** KEGG analysis of the relative abundance of metabolic pathways between *L. a. rubrogularis* and *L. a. vernayi*. The abscissa represents the relative abundance of functional pathways, the ordinate represents the functional pathways of the second classification level of KEGG, the rightmost box represents the first level to which the pathways belong. **(B)** Extended error bar plot to determine the significant difference in the predicted gene function between *L. a. rubrogularis* and *L. a. vernayi*. The middle value represents the mean differences between the groups (upper bar value – lower bar), and the error bar represents the 95% confidence intervals. The right side of the zero-point indicates the larger effect on the gene function in the *L. a. rubrogularis*, and the left side of the zero-point indicates the larger effect of that in the *L. a. vernayi*. *p* value at the side indicates the significance levels between the upper and lower bars. **(C)** Heatmap of the genus-level hierarchical clustering of the third level functional pathways in *L. a. rubrogularis* and *L. a. vernayi*.

According to the prediction, the KEGG Pathway Database classified metabolic pathways into six categories, including cellular processes, environmental information processing, genetic information processing, human diseases, metabolism, and organismal systems. Each metabolic pathway was further divided into several levels. The second level included 31 metabolic pathway sub-functions, and a total of 159 the third level functional pathways was predicted. Figure 6A demonstrates the average relative abundance of bacteria in all individuals of Silver-eared Mesia mapped to the secondary functional pathway of the KEGG database. The KEGG pathways showed that ‘metabolism’ was the most important pathway of gut microbiota.

Wilcoxon rank-sum test showed no significant difference in secondary metabolic pathways between *L. a. rubrogularis* and *L. a. vernayi* (Figure 6B) (secondary metabolic pathways with relative abundance of less than 1% in all samples were excluded and the *p*-values were corrected by Bonferroni). Clustering heat maps of the abundances of the top 40 third level functional pathways showed differences in functional abundances across samples, but the individuals of *L. a. rubrogularis* and *L. a. vernayi* were not clustered into two categories, respectively (Figure 6C). The results demonstrated no significant difference in the metabolic function of the intestinal microbiota between *L. a. rubrogularis* and *L. a. vernayi*. The LEfSe analysis was applied to all levels of KEGG metabolic pathways and also did not detect any variation between *L. a. rubrogularis* and *L. a. vernayi* (Supplementary Figure S2).

## Discussion

This is the first study to show the gut microbiota of Silver-eared Mesia whose habitat has been altered by trade. Previous studies have focused on comparing native habitats with captivity (Wienemann et al., 2011; Sun et al., 2019; Oliveira et al., 2020; San Juan et al., 2021; Grieves et al., 2022; Zhang et al., 2022). However, under captive conditions, the host’s living environment and diet are artificially controlled, so it is difficult to reflect the impact of the real field environment on the intestinal microbiota of immigrant species. In addition, under natural conditions, the host’s habitat environment and food sources are variable, such as the significant differences in diet composition between individuals within a species (Bodawatta et al., 2022b), which is difficult to overcome for cross-species and cross-regional comparison. In this study, native (*L. a. rubrogularis*) and immigrant (*L. a. vernayi*) subspecies lived in the same group, so their diet, environment, and other external factors were the same, and there were only slight phylogenetic differences, which can supply a unique opportunity to compare the gut microbiota of immigrant and native subspecies. Wildlife trade is a common route for introducing invasive species (Cardador et al., 2019). Traded migrant species may adversely affect native ecosystems, economic activity, and human well-being (Simberloff et al., 2013). Our findings can help to assess the risk of invasion of this non-native species for better understanding and management.

### The gut microbiota of immigrant subspecies coincides with the native subspecies

On the whole, our results demonstrated no significant differences in the composition, diversity, or functional metabolism of the gut microbiota between native and immigrant subspecies, which might be related to a similar diet, environment, and other external factors. The convergent evolution of gut microbiome, driven by similar dietary and environmental factors, has been found in other vertebrates as well. For example, the bamboo-eating *Ailuropoda melanoleuca* and *Ailurus styani* share more similarities in their gut microbiota structure and function with each other than their carnivorous relatives (Huang et al., 2021). Similarly, this phenomenon has been observed in studies of immigrants of different races. The gut microbiota of immigrants in the United States would become more and more similar to that of native people over time (Vangay et al., 2018; Peters et al., 2020; Copeland et al., 2021). To sum up, the intestinal microbiota of immigrant populations developed the same adaptive characteristics as that of native populations, and the generation of these adaptive characteristics may help immigrant populations adapt quickly to habitat changes (Alberdi et al., 2016).

### Characteristics of the gut microbiota of the Silver-eared Mesia living in mining area

Trace metals in the soil can be concentrated in the plants, which insects then feed on and carry trace elements into their bodies (Zhang et al., 2017). Thus, the extensive diet of Silver-eared Mesia will enrich a mass of trace elements into the body, making its intestinal microbiota under trace element stress environment. As a result, we also investigated the characteristics of its intestinal microbiota under trace elements stress. From the community composition of the gut microbes of native and immigrant subspecies, it was found that at the phylum level, *Proteobacteria* dominated the intestinal flora of the two subspecies (about 90%), followed by *Firmicutes*, *Bacteroidetes*, and *Actinobacteria*. Previous studies have found that *Firmicutes* are the main microbiome in the guts of mammals, chickens and wild birds (Waite and Taylor, 2014; Grond et al., 2018). Members of *Firmicutes* play an important role in the metabolism, digestion and absorption of proteins and other substances nutrients, and participate in the synthesis of digestive enzymes to assist the host in the digestion and absorption of nutrients (Grond et al., 2018). *Proteobacteria*, however, are mostly pathogenic bacteria, usually associated with intestinal ecological imbalances, metabolic and immune disorders (Colston and Jackson, 2016). Some studies of other wild birds have found *Firmicutes* to be the dominant species in the gut microbiota (Wang et al., 2017; Oliveira et al., 2020; Wu et al., 2021). In several other studies on wild birds, nevertheless, *Proteobacteria* were found to have a higher abundance than *Firmicutes* (Zhou et al., 2020; Zhang et al., 2022). Even so, compared with our study in which *Proteobacteria* accounted for almost 90% of the intestinal flora, *Proteobacteria* in their study were only slightly higher than *Firmicutes*. Given that the samples in these studies came from captivity or normal wild environments, it may be suggested that adaptation of intestinal flora to trace element stress may lead to *Proteobacteria* dominance. This is consistent with the characteristics of intestinal microbiota in other species under trace element stress (Šrut et al., 2019; Kakade et al., 2020; Wu et al., 2020).

### The role of intestinal microbiota in reducing heavy metal toxicity

Previous studies have shown that the gut microbiome is essential in protecting the host from the toxic effects of heavy metal exposure (Halttunen et al., 2008; Breton et al., 2013; Ninkov et al., 2015). In our study, we also found that the dominant group in the intestinal microbiome showed strong resistance to heavy metal toxicity. For instance, the *Pseudomonas* was dominant among almost all samples. *Pseudomonas* can degrade cellulose (Jiménez et al., 2014) and produce multitudinous antibiotic compounds, which can effectively stem the diseases caused by pathogenic bacteria and fungi (Haas and Défago, 2005). Particularly important, *Pseudomonas* showed a sky-high degree of resistance to heavy metals, such as Cd, Pb and As (Al-Ansari et al., 2021; Liu et al., 2021; Pramanik et al., 2021; Sun et al., 2021). In addition, several genera with high abundance, such as *Shigella*, *Vibrio* and *Halomonas*, also have high trace element resistance (Mukherjee et al., 2019; Jo et al., 2020; Bombaywala et al., 2021). These bacteria may play an important role in resisting trace element stress and maintaining normal growth, development and life activities of Silver-eared Mesia.

We also predicted the metabolic function of the gut microbiota of the Silver-eared Mesia. The metabolic pathways predicted by PICRUSt2 were consistent with those predicted by other wild species (Figure 6A; Zhou et al., 2020; Jiang F. et al., 2021; Zhang et al., 2022). Microbial metabolic pathways (such as xenobiotics biodegradation metabolism, carbohydrate metabolism, amino acid metabolism, and metabolism of cofactors and vitamins) accounted for more than 60% of the 31 predicted most abundant pathways. This suggests that the gut microbiome of Silver-eared Mesia may be involved in high levels of metabolic activity that may help to resist damage caused by trace elements.

### Speculation about when *L. a. vernayi* immigrated

We cannot evaluate the specific time of immigration of immigranted *L. a. vernayi*. According to our field survey results and some data (Jiang A. et al., 2021) in recent years, we can infer that its earliest migration time was around 2019, because it has not been found in the field survey before 2019. Interestingly, the Lefse analysis of our taxa may support this inference. Among all taxa of *L. a. rubrogularis* and *L. a. vernayi*, lefse analysis detected a genus, *Massilia*, with a significantly higher relative abundance in *L. a. vernayi* than that in *L. a. rubrogularis*. The *Massilia* are important resistant microbes in trace element stress environments, as it has been identified in mining soil, farmland soils, beach, and sludge polluted by trace elements (Feng et al., 2016; Krishnamoorthy et al., 2016; Lee et al., 2017; Wang et al., 2020). The *Massilia* can secrete a large amount of cyclodextrin, whose special physical structure can contain trace element ions (Santos and Barbosa-Tessmann, 2019), thus reducing the content of trace element in the intestinal environment and reducing its toxicity to Silver-eared Mesia (Badruddoza et al., 2013; Tajuddin Sikder et al., 2014). Another study on earthworms (*Eisenia fetida*) also found that *Massilia* showed a higher abundance after cadmium contamination, and that the abundance of *Massilia* decreased in the middle stage of cadmium pollution and increased to a stable level in the later stage (Zhou et al., 2021). This finding suggests that *L. a. vernayi* did not migrate for very long. We speculate that in the future, the abundance of *Massilia* in the intestinal tract of *L. a. vernayi* will fluctuate until it becomes similar to that of *L. a. rubrogularis*.

### Limitations

It should be noted that this study has several limitations. First, we do not understand the microbiome of the immigrant form, prior to our sampling. Secondly, we did not observe it in non-mining areas perhaps due to its rarity in China (second class national protected animal), which led to the inability to observe the adaptation process of the intestinal microbiota of Silver-eared Mesia under trace element stress. In addition, there are some differences in the sample numbers of *L. a. rubrogularis* and *L. a. vernayi*. Although the intestinal microbiota of *L. a. rubrogularis* and *L. a. vernayi* are very similar in our study, the differences in sample numbers may also cause some deviations in the results. From another perspective, however, the different sample numbers of the two subspecies may reflect their proportions in the wild-living taxa. There are far more immigrant subspecies than native ones, which could be detrimental to our biodiversity conservation. We suggest that some controlled experiments in non-mining areas and the habitat of *L. a. vernayi* should be carried out in future studies, which will be of significance for the protection of wild animals.

## Conclusion

In conclusion, the composition, diversity, and function of the intestinal microbiota of immigrant subspecies (*L. a. vernayi*) were basically the same as those of the native subspecies (*L. a. rubrogularis*). The gut microbiota of the Silver-eared Mesia living in the mining area developed adaptive characteristics to trace element stress. The significance of this study lies in: (1) the results shed some light on the adaptation of the gut microbiota of immigrant species to cope with habitat changes after “migration”. It supplies new insights into the adaptation mechanism of gut microbiota in response to environmental changes in wild animals, thus providing important implications for wildlife conservation and biodiversity conservation. (2) The results also showed the adaptation mechanism of the intestinal microbiota of birds under trace element environmental stress (how to change the composition and function of the microbiota to resist the toxic effects of trace elements). It is helpful to understand the toxicity of trace elements to birds and the strategies of birds to adapt to trace element pollution, which has a guiding role in pollution control and biodiversity protection in mining areas.

## Data availability statement

The datasets presented in this study can be found in online repositories. The names of the repository/repositories and accession number(s) can be found at: https://www.ncbi.nlm.nih.gov/, PRJNA853520.

## Ethics statement

The animal study was reviewed and approved by Chongzuo Forestry Bureau and Ethics Committee of Guangxi University.

## Author contributions

TZ, SL, and AJ contributed intellectual input and assistance to this study. TZ and AJ designed the research. TZ and SL did the sampling and laboratory work. TZ conducted data analysis and wrote the first draft of the manuscript. AJ contributed substantially to revisions. All authors contributed to the article and approved the submitted version.

## Funding

This work was funded by the National Natural Science Foundation of China (31870370) and the Key Grant of Guangxi Nature and Science Foundation (2018GXNSFDA281016).

## Conflict of interest

The authors declare that the research was conducted in the absence of any commercial or financial relationships that could be construed as a potential conflict of interest.

## Publisher’s note

All claims expressed in this article are solely those of the authors and do not necessarily represent those of their affiliated organizations, or those of the publisher, the editors and the reviewers. Any product that may be evaluated in this article, or claim that may be made by its manufacturer, is not guaranteed or endorsed by the publisher.

## Footnotes

^1^ https://birdsoftheworld.org

^2^ https://docs.qiime2.org/2022.2/tutorials/

^3^ https://www.kegg.jp/

^4^ http://circos.ca/
