# Supplementary material for: Comparison of gut microbiota between immigrant and native populations of the Silver-eared Mesia (Leiothrix argentauris) living in mining area
Source: Front Microbiol. 2023 Jan 24;14:1076523. doi: 10.3389/fmicb.2023.1076523 (PMC9904241; doi:10.3389/fmicb.2023.1076523)
Supplement: Supplementary file 1 [file Data_Sheet_1.docx]

Supplementary Material

**Part 1 | Supplementary Figures**

**Supplementary Figures**


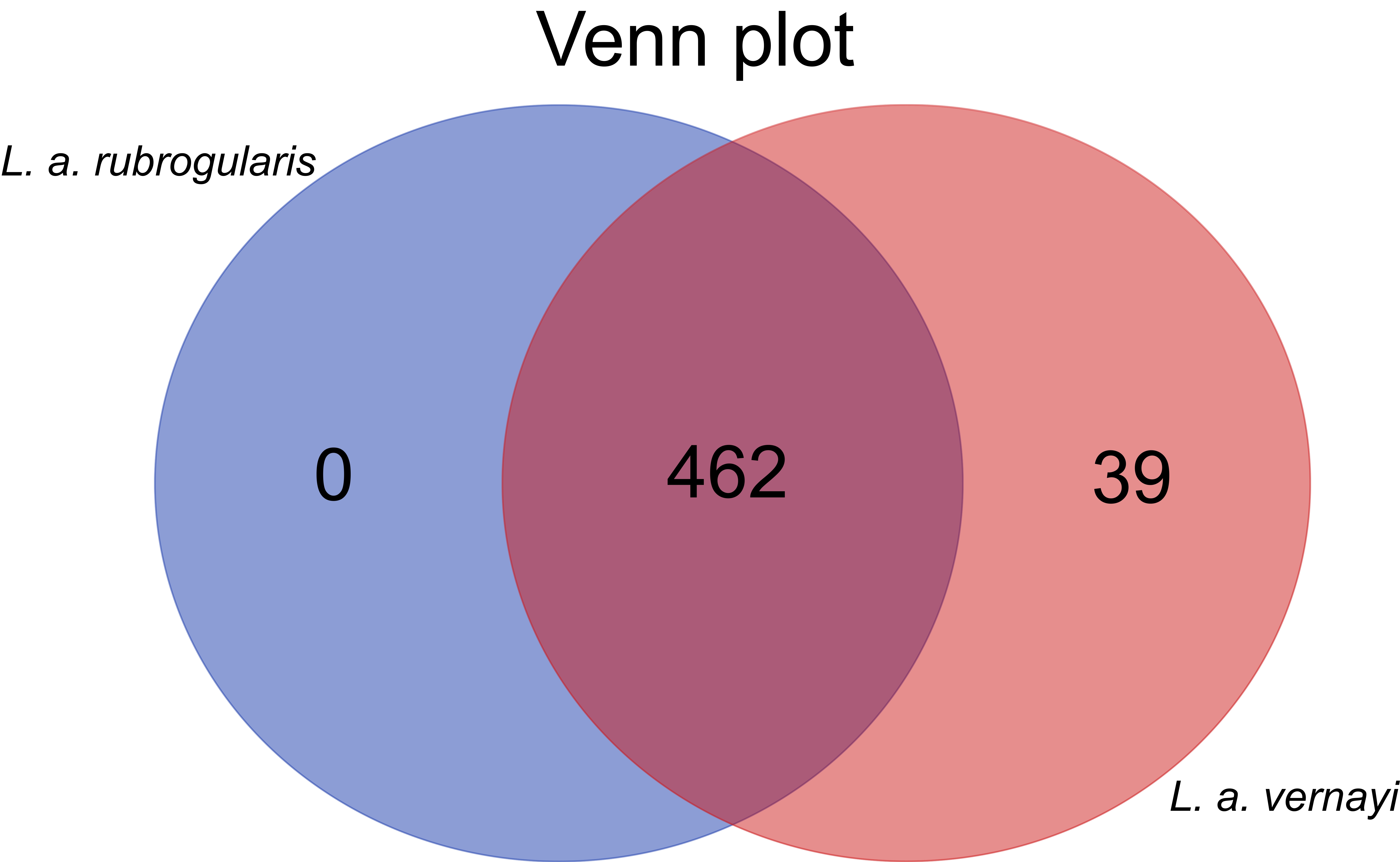


**Supplementary Figure 1 |** Venn diagram of shared and unique OTUs in the gut microbiota between *L. rubrogularis* and *L. vernayi*.

**
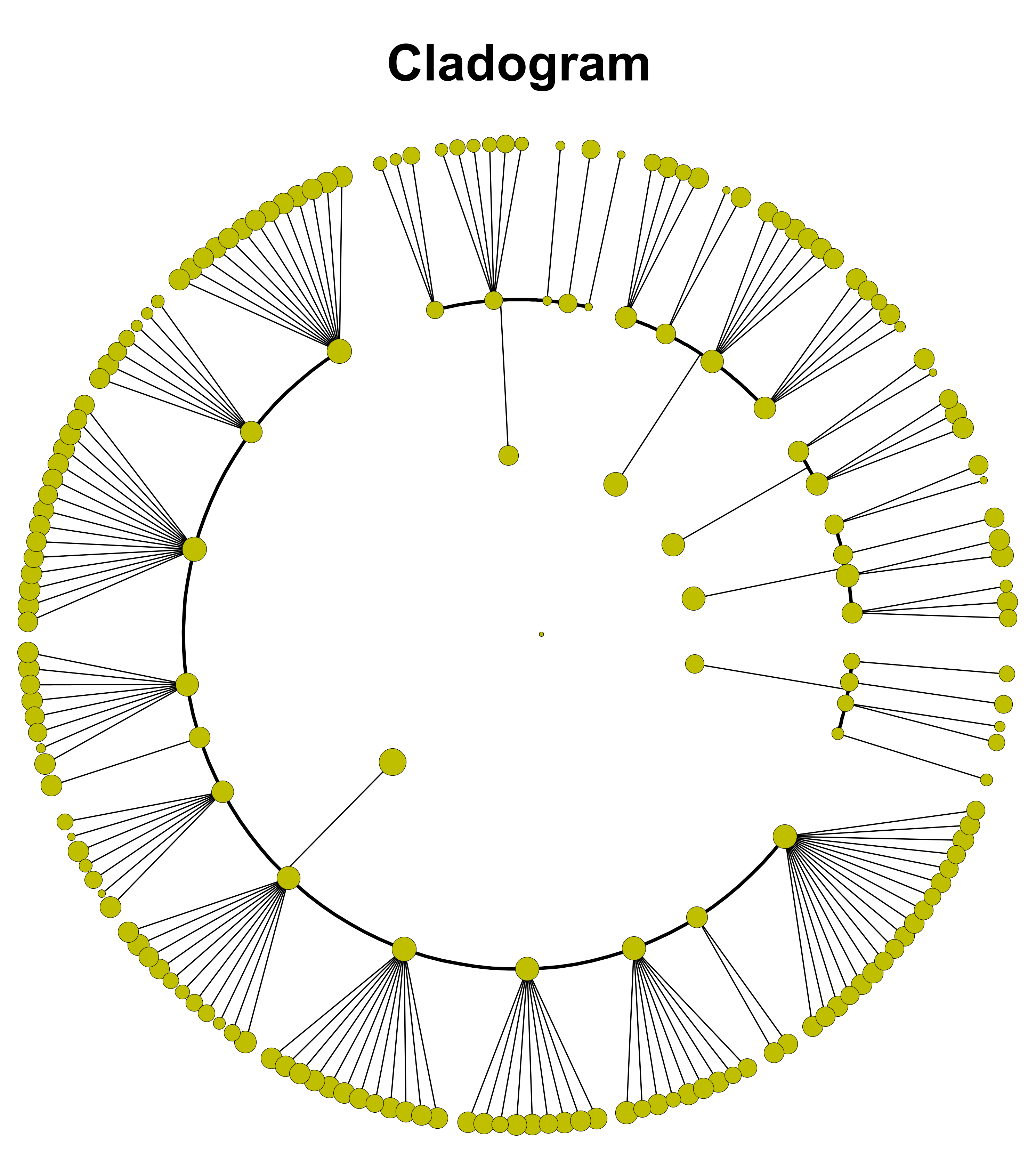
**

**Supplementary Figure 2 |** LEfSe analysis of the metabolic pathways between *L. rubrogularis* and *L. vernayi*.

**Supplementary Material**

**Part 2 | MATERIALS AND METHODS**

**Measurement of trace metal content**

Prior to analysis, the feathers were vigorously rinsed alternately with ultra-pure water and acetone to remove adhesion external contamination (Liu et al., 2022). After washing, put them into the oven and dry them at 60℃ for 24 h (Dauwe et al., 2003). Dry weight of all feather samples was determined using a digital balance. Then, feather samples were digested in clean Teflon tubes with 6ml 65% (GR grade) HNO_3_ using a graphite digestion system (Abdullah et al., 2015). The solutions were diluted to 25ml with ultrapure water, filtered, and stored in tubes ready for trace metals analysis (Abdullah et al., 2015). Finally, five elements (As, Cd, Pb, Cu and Zn) were measured using Inductively Coupled Plasma Optical Emission Spectrometry (ICP-OES; model ICP-5000) at the College of Agriculture, Guangxi University.

**REFERENCES**

Abdullah, M., Fasola, M., Muhammad, A., Malik, S.A., Bostan, N., Bokhari, H., et al. (2015). Avian feathers as a non-destructive bio-monitoring tool of trace metals signatures: A case study from severely contaminated areas. *Chemosphere* 119**,** 553-561. doi: 10.1016/j.chemosphere.2014.06.068

Dauwe, T., Bervoets, L., Pinxten, R., Blust, R., and Eens, M. (2003). Variation of heavy metals within and among feathers of birds of prey: effects of molt and external contamination. *Environ Pollut* 124**,** 429-436. doi: 10.1016/S0269-7491(03)00044-7

Liu, S., Mtemi, W.M., Zhou, T., Pan, J., and Jiang, A. (2022). Multiple trace elements exposure of Grey-cheeked Fulvettas *Alcippe morrisonia*, a nuclear member in bird mixed-species flocks, and implications for bioindicator. *Ecotoxicol. Environ. Saf.* 244**,** 114063. doi: 10.1016/j.ecoenv.2022.114063
